# Evolution of retinoic acid receptors in chordates: insights from three lamprey species, *Lampetra fluviatilis*, *Petromyzon marinus*, and *Lethenteron japonicum*

**DOI:** 10.1186/s13227-015-0016-4

**Published:** 2015-05-07

**Authors:** Florent Campo-Paysaa, David Jandzik, Yoko Takio-Ogawa, Maria V Cattell, Haley C Neef, James A Langeland, Shigeru Kuratani, Daniel M Medeiros, Sylvie Mazan, Shigehiro Kuraku, Vincent Laudet, Michael Schubert

**Affiliations:** Molecular Zoology Team, Institut de Génomique Fonctionnelle de Lyon, Université de Lyon, Université Lyon 1, CNRS, INRA, Ecole Normale Supérieure de Lyon, 69364 Lyon Cedex 07, France; MRC Centre for Developmental Neurobiology, New Hunt’s House, King’s College London, Guy’s Campus, London, SE1 1UL UK; Department of Ecology and Evolutionary Biology, University of Colorado Boulder, Ramaley Biology, 1800 Colorado Avenue, Boulder, CO 80309 USA; Department of Zoology, Comenius University in Bratislava, Mlynska Dolina B-1, 84215 Bratislava, Slovakia; Laboratory for Evolutionary Morphology, RIKEN, 2-2-3 Minatojima-minamimachi, Chuo-ku, Kobe, Hyogo 650-0047 Japan; Department of Pediatrics, University of Colorado, Children’s Hospital, 13065 East 17th Avenue, Aurora, CO 80045 USA; Department of Biology, Kalamazoo College, 1200 Academy Street, Kalamazoo, Michigan 49008 USA; Division of Pediatric Gastroenterology, Department of Pediatrics and Communicable Diseases, University of Michigan, C.S. Mott Children’s Hospital, 1540 East Hospital Drive SPC 4259, Ann Arbor, Michigan 48109 USA; Sorbonne Universités, UPMC Université Paris 06, FR2424, Station Biologique de Roscoff, Place Georges Teissier, 29680 Roscoff, France; CNRS, FR2424, Station Biologique de Roscoff, Place Georges Teissier, 29680 Roscoff, France; Genome Resource and Analysis Unit, RIKEN Center for Developmental Biology, 2-2-3 Minatojima-minamimachi, Chuo-ku, Kobe, Hyogo 650-0047 Japan; Phyloinformatics Unit, RIKEN Center for Life Science Technologies, 2-2-3 Minatojima-minamimachi, Chuo-ku, Kobe, Hyogo 650-0047 Japan; Sorbonne Universités, UPMC Université Paris 06, UMR 7009, Laboratoire de Biologie du Développement de Villefranche-sur-Mer, Observatoire Océanologique de Villefranche-sur-Mer, 181 Chemin du Lazaret, 06230 Villefranche-sur-Mer, France; CNRS, UMR 7009, Laboratoire de Biologie du Développement de Villefranche-sur-Mer, Observatoire Océanologique de Villefranche-sur-Mer, 181 Chemin du Lazaret, 06230 Villefranche-sur-Mer, France

**Keywords:** Agnathan, Cyclostome, Developmental patterning, Gene duplication, Gnathostome, RAR code, Retinoid signaling, Vertebrate

## Abstract

**Background:**

Retinoic acid (RA) signaling controls many developmental processes in chordates, from early axis specification to late organogenesis. The functions of RA are chiefly mediated by a subfamily of nuclear hormone receptors, the retinoic acid receptors (RARs), that act as ligand-activated transcription factors. While RARs have been extensively studied in jawed vertebrates (that is, gnathostomes) and invertebrate chordates, very little is known about the repertoire and developmental roles of RARs in cyclostomes, which are extant jawless vertebrates. Here, we present the first extensive study of cyclostome RARs focusing on three different lamprey species: the European freshwater lamprey, *Lampetra fluviatilis*, the sea lamprey, *Petromyzon marinus*, and the Japanese lamprey, *Lethenteron japonicum*.

**Results:**

We identified four *rar* paralogs (*rar1*, *rar2*, *rar3*, and *rar4*) in each of the three lamprey species, and phylogenetic analyses indicate a complex evolutionary history of lamprey *rar* genes including the origin of *rar1* and *rar4* by lineage-specific duplication after the lamprey-hagfish split. We further assessed their expression patterns during embryonic development by *in situ* hybridization. The results show that lamprey *rar* genes are generally characterized by dynamic and highly specific expression domains in different embryonic tissues. In particular, lamprey *rar* genes exhibit combinatorial expression domains in the anterior central nervous system (CNS) and the pharyngeal region.

**Conclusions:**

Our results indicate that the genome of lampreys encodes at least four *rar* genes and suggest that the lamprey *rar* complement arose from vertebrate-specific whole genome duplications followed by a lamprey-specific duplication event. Moreover, we describe a combinatorial code of lamprey *rar* expression in both anterior CNS and pharynx resulting from dynamic and highly specific expression patterns during embryonic development. This ‘RAR code’ might function in regionalization and patterning of these two tissues by differentially modulating the expression of downstream effector genes during development.

**Electronic supplementary material:**

The online version of this article (doi:10.1186/s13227-015-0016-4) contains supplementary material, which is available to authorized users.

## Background

Developmental functions of vitamin A derivatives (also called retinoids) have been described in great detail in various vertebrate species since the first half of the twentieth century [1]. Although a variety of retinoids are detectable in vertebrates [2], all-*trans* retinoic acid (RA) is the main biologically active vitamin A derivative during embryogenesis [3-5]. RA signaling is involved in the control of a wide range of biological processes. In the course of vertebrate development, for example, RA controls cell proliferation, cell differentiation, apoptosis, and cell survival, acting at different developmental stages, from early gastrulation to late organogenesis, and in all embryonic tissue layers. Roles for RA signaling during development have also been characterized in invertebrate chordates, that is, in cephalochordates and tunicates, and it has been shown that RA functions, in particular in developmental patterning, are well conserved, at least between cephalochordates and vertebrates [3].

The molecular response to RA is controlled by heterodimers of two members of the nuclear hormone receptor superfamily: the retinoic acid receptor (RAR) and the retinoid X receptor (RXR) [6-9]. According to the classical model of RAR/RXR heterodimer function, unliganded heterodimers exert a repressive action on the expression of their targets, while, upon RA binding, the heterodimers recruit co-activators and promote the transcription of target genes [6,9]. RAR/RXR target gene specificity is mediated by the binding of the heterodimer to specific DNA elements in the regulatory regions of target genes, the so-called retinoic acid response elements (RAREs) [10-13].

In vertebrates, whole genome duplication (WGD) events led to the expansion of the repertoire of *rar* and *rxr* genes [14]. Thus, while the cephalochordate amphioxus possesses only one *rar* and one *rxr*, the mouse genome encodes three *rar* genes and three *rxr* genes, called *rarα*, *rarβ*, and *rarγ*, and *rxrα*, *rxrβ*, and *rxrγ*, respectively. The *rar* and *rxr* repertoires have been expanded further in some bony fish (the teleosts), whose genomes have undergone an additional round of WGD [15-17]. Thus, the zebrafish genome encodes four *rar* genes (*rarαa*, *rarαb*, *rarγa*, and *rarγb*) and six *rxr* genes (*rxrαa*, *rxrαb*, *rxrβa*, *rxrβb*, *rxrγa*, and *rxrγb*). During chordate development, expression of *rar* and *rxr* genes is generally dynamic and detectable in most embryonic tissues [16-21]. Interestingly, paralogous *rar* and *rxr* genes in jawed vertebrates (that is, gnathostomes) show highly diverse expression patterns as well as divergent functions during development, indicating that vertebrate-specific genome duplications have mediated lineage-specific diversification of the developmental processes controlled by specific *rar* and *rxr* genes [17,21].

Surprisingly, while developmental roles of RA signaling have been extensively studied in gnathostomes and invertebrate chordates, much less is known about RA functions in cyclostomes [22-24], a group of jawless vertebrates comprising lampreys and hagfish and representing the phylogenetic sister group of the gnathostomes [25]. Cyclostomes are particularly appealing models for comparative studies, because they possess many vertebrate-specific features, such as neural crest derivatives, but lack key characters that are present in other vertebrates, such as the jaws [26-28]. In addition to the overall morphology, cyclostome genomes, when compared to those of gnathostomes, also exhibit both similarities and differences [29]. For instance, while lamprey genomes have very likely experienced the two rounds of WGDs characteristic of vertebrates [30-32], their genomes undergo dramatic remodeling during development, resulting in the elimination of hundreds of millions of base pairs (bp), including hundreds of genes, from somatic cell lineages [33,34].

In lampreys, some preliminary studies have been carried out to investigate the roles of RA signaling during embryonic development and have provided insights into the evolution of RA functions in the vertebrate lineage [22-24]. For example, it has been shown that RA treatments during gastrulation induce rostral truncations of both the brain and the pharynx, leading, in the severest cases, to embryos that consist only of trunk segments [22]. Previous work has also suggested that the genomes of the sea lamprey, *Petromyzon marinus*, the Japanese lamprey, *Lethenteron japonicum*, the Australian lamprey, *Mordacia mordax*, as well as of the inshore hagfish, *Eptatretus burgeri*, encode at least three *rar* genes [24,31], although phylogenetic analyses have failed to unambiguously assign orthologies between the cyclostome and gnathostome *rar*s [31]. Furthermore, the expression patterns of lamprey *rar*s have so far only been described for a single developmental stage, and the functions of these genes in the lamprey embryo still remain elusive [24].

Given the lack of data about the developmental expression and the functions of lamprey *rar* genes, we decided to isolate and characterize the *rar* genes from a third lamprey species, the European river lamprey (*Lampetra fluviatilis*). We identified and cloned cDNAs of four *L. fluviatilis rar* genes and investigated their phylogenetic relationship to *rar*s of other vertebrates, including two additional lamprey species (*P. marinus* and *L. japonicum*). Furthermore, we carefully characterized the expression of the four *L. fluviatilis rar* genes in the course of embryogenesis and compared the obtained patterns to those of the *P. marinus* and *L. japonicum rar* genes. Our results indicate that lamprey genomes encode at least four *rar* genes, including two resulting from a lamprey-specific duplication event. Intriguingly, analyses of the developmental expression of the *L. fluviatilis*, *P. marinus*, and *L. japonicum rar* genes reveal dynamic and partially overlapping gene expression patterns in both central nervous system (CNS) and pharynx. The *rar* expression domains in these embryonic tissues seem to establish a combinatorial ‘RAR code,’ which might function in patterning and regionalization of the lamprey CNS and pharynx. Taken together, this work provides the first detailed description of *rar* expression during cyclostome development and reveals fundamental information on the elaboration of RA signaling functions following duplication of an ancestral *rar* gene early in vertebrate evolution.

## Methods

### Embryos

Adult male and female *L. fluviatilis*, *P. marinus*, and *L. japonicum* were collected, respectively, from the Loire river (France), from tributaries of Lake Huron and Lake Michigan (USA), and from the Miomote and Shiribetsu rivers (Japan). Collection and handling of animals was carried out in full compliance of institutional, national, and international guidelines and did not require approval by an ethics committee. After stripping the adults, eggs were artificially fertilized and incubated in filtered water at 12°C (for *L. fluviatilis*), in 0.1× Marc’s modified Ringer’s (MMR) buffer [24] at 18°C (for *P. marinus*), or in 10% Steinberg’s solution [35] at 16°C to 23°C (for *L. japonicum*). Embryonic stages were assessed morphologically according to the developmental table for *L. reissneri* [36]. For *in situ* hybridization and immunohistochemistry analyses, the embryos were fixed in 4% paraformaldehyde (PFA) in phosphate-buffered saline (PBS), dehydrated in a methanol dilution series, and stored in 100% methanol at −20°C.

### Gene isolation, cloning, and sequencing

The clones of *L. fluviatilis rar*s were isolated from cDNA libraries and by polymerase chain reaction (PCR) amplification. Sequences of the primers used for PCR experiments are available from the authors. Template cDNA was prepared from total RNA extracted from pooled *L. fluviatilis* embryos of stages 22 through 26 using the First-Strand cDNA Synthesis Kit (GE Healthcare, Velizy-Villacoublay, France). PCR products were purified with the QIAquick PCR Purification Kit (Qiagen, Courtaboeuf, France) and then cloned into the pCRII-TOPO vector (Invitrogen, Cergy Pontoise, France). After cloning, the obtained *L. fluviatilis rar* gene fragments were sequenced on both strands (Cogenics, Meylan, France). Amplification of 5′ and 3′ regions of the original cDNA clones was carried out by 5′ and 3′ rapid amplification of cDNA ends (RACE) using the GeneRacer Advanced RACE Kit (Invitrogen, Cergy Pontoise, France). After cloning, the obtained 5′ and 3′ RACE fragments were sequenced on both strands (Cogenics, Meylan, France). Altogether, the cloning yielded the following *L. fluviatilis rar* gene pieces: *rar1* (length 1,116 bp, GenBank accession number KJ948416), *rar2* (length 1,104 bp, GenBank accession number KJ948417), *rar3* (length 1,475 bp, GenBank accession number KJ948418), and *rar4* (length 1,071 bp, GenBank accession number KJ948419). Additionally, the genome sequences of both *P. marinus* [32] and *L. japonicum* [37] were systematically screened for the presence of *rar* genes, which led to the identification of the *P. marinus* and *L. japonicum rar1*, *rar2*, *rar3*, and *rar4* genes, which, with the exception of *L. japonicum rar4*, were subsequently validated by cloning and sequencing, as previously described [24,31]. The GenBank accession numbers of the *P. marinus* and *L. japonicum rar1*, *rar2*, *rar3*, and *rar4* genes are as follows: *P. marinus rar1*, LC019144 (length 576 bp); *P. marinus rar2*, LC019145 (length 1,378 bp); *P. marinus rar3*, LC019146 (length 1,247 bp); *P. marinus rar4*, LC019147 (length 1,050 bp); *L. japonicum rar1*, AB292622 (length 1,842 bp); *L. japonicum rar2*, AB292623 (length 2,753 bp); *L. japonicum rar3*, AB292624 (length 2,307 bp); *L. japonicum rar4*, LC019148 (length 1,077 bp).

### Sequence analysis and phylogenetic reconstruction

The deduced protein sequences encoded by the *rar* genes from various animals, including the three lamprey species analyzed in this study, were aligned using MAFFT [38] followed by manual refinement (the alignment is available from the authors upon request), and the phylogenetic tree was inferred with the maximum likelihood (ML) method using PhyML version 3.0 [39] based on 144 gap-free amino acid sites in the alignment. The phylogenetic inference employed the LG + I + Γ_4_ model of amino acid substitution and bootstrap resampling (1,000 replicates).

### Whole mount *in situ* hybridization

Digoxigenin (DIG)-labeled antisense and sense riboprobes of the four *L. fluviatilis* and *P. marinus rar* genes as well as of the three cloned *L. japonicum rar* genes were transcribed using DIG-11-UTP (Roche, Boulogne-Billancourt, France) according to the manufacturer’s instructions. The *in situ* hybridization experiments for *P. marinus* and *L. japonicum* were performed as previously described [24,40]. For *L. fluviatilis*, fixed embryos were rehydrated in PBS containing 0.1% Tween 20 (PBT). After rehydration, the embryos were digested for 30 min in 10 μg/ml proteinase K (Sigma-Aldrich, Saint-Quentin Fallavier, France) in PBT. Subsequently, the embryos were post-fixed for 15 min with PFA/PBT containing 0.2% glutaraldehyde, then washed with PBT and pre-hybridized in hybridization buffer (50% formamide, 1.3× SSC, 0.2% Tween 20, 50 μg/ml total yeast RNA, 100 μg/ml heparin sulfate, 5 mM ethylenediaminetetraacetic acid (EDTA)-Na_2_, 0.5% CHAPS) for 2 h at 72°C. The specimens were then incubated in hybridization buffer with 0.1 mg/ml DIG-labeled RNA probe overnight at 72°C. After hybridization, the embryos were washed four times in hybridization buffer for 30 min at 72°C. Subsequently, the solution was substituted gradually with 10 mM Tris–HCl (pH 8) containing 0.5 M NaCl and 1 mM EDTA (NTE). RNase A was added to a final concentration of 100 μg/ml, and the specimens were incubated for 30 min at 37°C. The samples were then washed twice with hybridization buffer for 30 min at 65°C and once in 50% hybridization buffer and 50% maleic acid buffer (pH 7.5) with 0.1% Tween 20 (MABT) for 30 min at 65°C. The embryos were blocked with MABT containing 0.5% blocking reagent (Roche, Boulogne-Billancourt, France) and 20% sheep serum for 2 h and developed at 4°C overnight with alkaline phosphatase (AP)-conjugated anti-digoxigenin Fab fragments, diluted 1:2,000 (Roche, Boulogne-Billancourt, France). The embryos were washed ten times for 30 min each in MABT at room temperature and then overnight at 4°C. Subsequently, the embryos were washed twice in 100 mM NaCl, 100 mM Tris (pH 9.6), 50 mM MgCl_2_, and 0.1% Tween 20 for 15 min at room temperature. Alkaline phosphatase activity was detected with BM Purple (Roche, Boulogne-Billancourt, France). Stained specimens were fixed in 4% PFA in PBS, dehydrated with a methanol series and clarified with a 1:2 mixture of benzyl alcohol and benzyl benzoate.

### Histology

After *in situ* hybridization, embryos were post-fixed in 4% PFA/PBS (at 4°C, overnight), rinsed in PBS, dehydrated in methanol (25%, 50%, 75%, 100%, and 100%), and incubated in methylcyclohexane. *L. fluviatilis* embryos were subsequently embedded in paraplast and sectioned at 10 μm with a microtome, while *P. marinus* and *L. japonicum* embryos were embedded in gelatin and sectioned at 10 μm using a cryostat [24,41].

### Whole mount immunohistochemistry

The fixed embryos stored in 100% methanol were placed in dimethylsulfoxide (DMSO) and methanol (1:1). After washes with Tris-buffered saline (pH 7.6) with 0.1% Tween 20 (TST) containing 5% DMSO (TSTd), the embryos were blocked with aqueous 1% periodic acid and 5% nonfat dry milk in TSTd (TSTM). The specimens were incubated for 4 days at room temperature with antibodies directed against acetylated tubulin (Sigma-Aldrich, Saint-Quentin Fallavier, France) diluted 1:1,000 in TSTM. After incubation, the samples were washed with TST and incubated with HRP-conjugated anti-mouse IgG antibodies, diluted 1:200 in TSTM (Invitrogen, Cergy Pontoise, France). After the final wash in TSTd, the embryos were developed with the peroxidase substrate 3,3′-diaminobenzidine (DAB) at 100 mg/ml in TST for 1 h and subsequently with DAB at 100 mg/ml in TST in the presence of 0.01% hydrogen peroxide.

## Results and discussion

### Lamprey genomes encode at least four *rar* genes

Previous analyses have identified three *rar* genes (called *rar1*, *rar2*, and *rar3*) in the genomes of three different lamprey species, *P. marinus*, *L. japonicum*, and *M. mordax* [24,31]. Lamprey *rar3* was shown to group with the gnathostome *rarα* genes, while *rar2* and *rar1* could tentatively be associated, respectively, with the gnathostome *rarβ* and *rarγ* genes [31]. However, the statistical support for this phylogenetic arrangement was not very strong [31]. In this study, we assessed the *rar* complement of the European river lamprey, *L. fluviatilis*, and successfully identified and cloned four *rar* genes from this lamprey species. The newly characterized fourth *rar* gene was subsequently identified in the genomes of both *P. marinus* [32] and *L. japonicum* [37] and validated in *P. marinus* by PCR-based cloning from cDNA. Following the logic of the nomenclature used in previous studies on lamprey *rar* genes, this novel, fourth, lamprey *rar* gene was named *rar4*.

**Figure 1:**
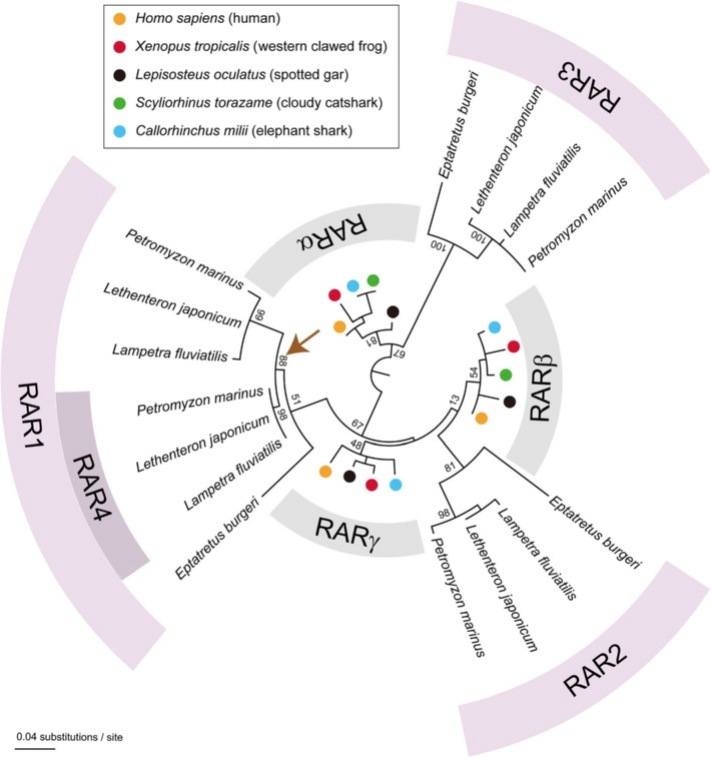
Molecular phylogeny of cyclostome retinoic acid receptors (RARs). The maximum likelihood tree was inferred with 144 amino acid sites and visualized using FigTree (version 1.4.2), which specified the root of the tree using the midpoint root function. Bootstrap support values (percentages ranging from 0 to 100) are indicated only for crucial nodes. The arrow highlights the position of the gene duplication giving rise to the lamprey *rar1* and *rar4* genes.

We subsequently carried out a phylogenetic analysis to address the relationships between the four RARs of *L. fluviatilis*, *P. marinus*, and *L. japonicum* and those from other animals. Consistent with previously published data [31], the resulting phylogeny recovered the proximities of gnathostome RARα and cyclostome RAR3 as well as of gnathostome RARβ and cyclostome RAR2 (Figure 1). In contrast, our analysis did not retrieve the relationship of gnathostome RARγ and cyclostome RAR1 (Figure 1). More importantly, in our phylogenetic tree, the lamprey RAR1 and RAR4 sequences grouped together at the exclusion of the RAR1 from the inshore hagfish (*E. burgeri*), strongly suggesting that *rar1* and *rar4* arose by gene duplication in the lineage leading to extant lampreys after the hagfish-lamprey split (Figure 1).

Taken together, our phylogenetic data are compatible with the scenario that lamprey *rar* genes have undergone both pan-vertebrate and lamprey-specific gene duplications. The lamprey *rar*s thus likely belong to the three gnathostome subtypes (that is, *rarα*, *rarβ*, and *rarγ*), but further bioinformatic analyses will be required to validate the proposed associations of lamprey and gnathostome *rar* paralogs. Although it was initially believed that cyclostomes (that is, lampreys and hagfish) might have diverged from other vertebrates (that is, gnathostomes) before the second round of WGD in the vertebrate lineage [42], more recent publications propose instead that cyclostomes may have also undergone the two rounds of WGD [30-32]. Our data on the phylogenetic relationships of lamprey *rar* genes are in agreement with these latest hypotheses on the timing of WGDs in the vertebrate lineage. Moreover, it has also been suggested that a significant number of gene duplications have occurred specifically in the lineage leading to extant lampreys. For instance, comparative analyses of vertebrate *hox* gene repertoires have identified lineage-specific *hox* cluster duplications in lampreys [37,43-45]. With the description of two *rarγ* subtype genes in lampreys, *rar1* and *rar4*, our work adds another example to the list of genes that underwent a lineage-specific duplication in lampreys.

### The lamprey *rar*s are expressed in specific spatiotemporal domains during embryonic development

Following the phylogenetic analysis of lamprey *rar* genes, we assessed the temporal and spatial expression patterns of these genes during development. We thus characterized the expression of the four *L. fluviatilis rar*s (*rar1*, *rar2*, *rar3*, and *rar4*) by *in situ* hybridization at various stages of embryonic development and carefully mapped the obtained signals using as landmarks the expression of marker genes in specific brain regions as well as the immunohistochemical signature of neurons in the developing head (Additional file 1: Figure S1 and Additional file 2: Figure S2). Additionally, we assessed the developmental expression patterns of the four *P. marinus rar*s (*rar1*, *rar2*, *rar3*, and *rar4*) as well as of three of the four *L. japonicum rar*s (*rar1*, *rar2*, and *rar3*) to allow comparisons of *rar* expression between the different lamprey species. For studying the developmental patterns of the four *L. fluviatilis* and *P. marinus rar* genes, we focused on embryos from stages 19 (that is, neurula) through 26 (that is, body elongation), and, for analysis of the domains of the three *L. japonicum rar* genes, we used embryos at body elongation stages 24 through 26 [36].

### Expression of lamprey *rar1* and *rar4* genes during development

**Figure 2:**
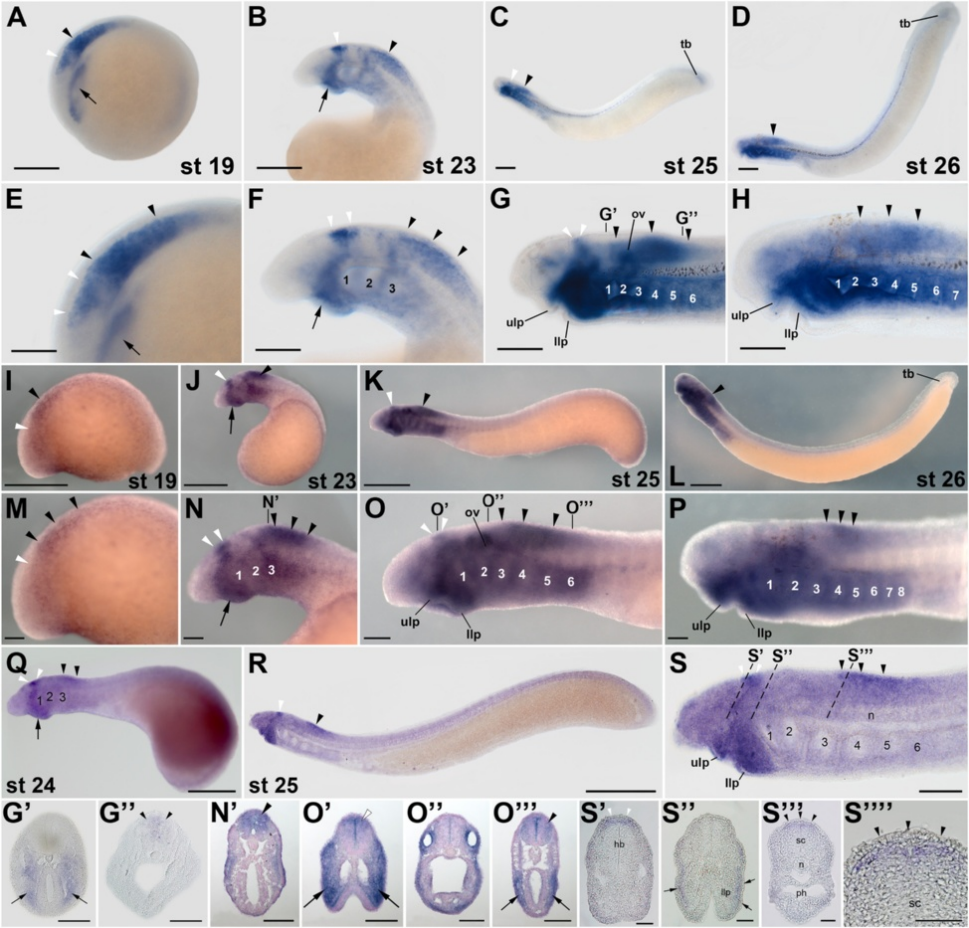
Expression of the retinoic acid receptor (*rar*) gene *rar1* during development of the European river lamprey, *Lampetra fluviatilis*, of the sea lamprey, *Petromyzon marinus*, and of the Japanese lamprey, *Lethenteron japonicum*. The expression of *L. fluviatilis rar1* was characterized by *in situ* hybridization in embryos at stages (st) 19 **(A)** and **(E)**, 23 **(B)** and **(F)**, 25 **(C)** and **(G)**, as well as 26 **(D)** and **(H)**. Higher magnifications of the anterior regions of embryos at stages 19, 23, 25, and 26 are shown in **(E)**, **(F)**, **(G),** and **(H)**, respectively. Cross sections of the embryo at stage 25 are midway through the hindbrain **(G’)** and through the anterior spinal cord **(G”)**. The expression of *P. marinus rar1* was characterized by *in situ* hybridization in embryos at stages 19 **(I)** and **(M)**, 23 **(J)** and **(N)**, 25 **(K)** and **(O)**, as well as 26 **(L)** and **(P)**. Higher magnifications of the anterior regions of embryos at stages 19, 23, 25, and 26 are shown in **(M)**, **(N)**, **(O)**, and **(P)**, respectively. The cross section of the embryo at stage 23 is through the anterior spinal cord **(N’)** and those of the embryo at stage 25 are through the mouth region **(O’)**, otic vesicle **(O”)**, and posterior pharynx **(O''')**. The expression of *L. japonicum rar1* was analyzed by *in situ* hybridization in embryos at stages 24 **(Q)** as well as 25 **(R)** and **(S)**. A higher magnification of the anterior region of the embryos at stage 25 is shown in **(S)**. Cross sections of the embryo at stage 25 are through the hindbrain **(S’)**, mouth region **(S”)**, and anterior spinal cord **(S''')**. The exact anteroposterior levels of the sections shown in **(S’)**, **(S”)**, and **(S”’)** are indicated by dotted lines in **(S)**. **(S'''')** shows a magnification of the anterior spinal cord in **(S”’)**. All embryos are oriented with anterior to the left. White arrowheads indicate hindbrain expression and black arrowheads expression in the spinal cord. Arrows in **(A)** and **(E)** as well as in **(G’)** and **(O”’)** show pharyngeal expression, while arrows in **(B)**, **(F)**, **(J)**, **(N)**, **(Q)**, as well as in **(O’)** and **(S”)** highlight mandibular expression. Scale bars for *L. fluviatilis*: 100 μm for **(A)**, **(B)**, **(C)**, and **(D)**; 50 μm for **(E)**, **(F)**, **(G)**, **(H)**, **(G’)**, and **(G”)**. Scale bars for *P. marinus*: 500 μm for **(I)**, **(J)**, **(K)**, and **(L)**; 100 μm for **(M)**, **(N)**, **(O)**, **(P)**, **(N’), (O’)**, **(O”)**, and **(O''')**. Scale bars for *L. japonicum*: 500 μm for **(Q)**; 1,000 μm for **(R)**; 100 μm for **(S)**; 50 μm for **(S’)**, **(S”)**, **(S”’)**, and **(S'''')**. Abbreviations: hb, hindbrain; llp, lower lip; n, notochord; ov, otic vesicle; ph, pharynx; sc, spinal cord; tb, tail bud; ulp, upper lip; 1 to 8, pharyngeal pouches 1 to 8.

**Figure 3:**
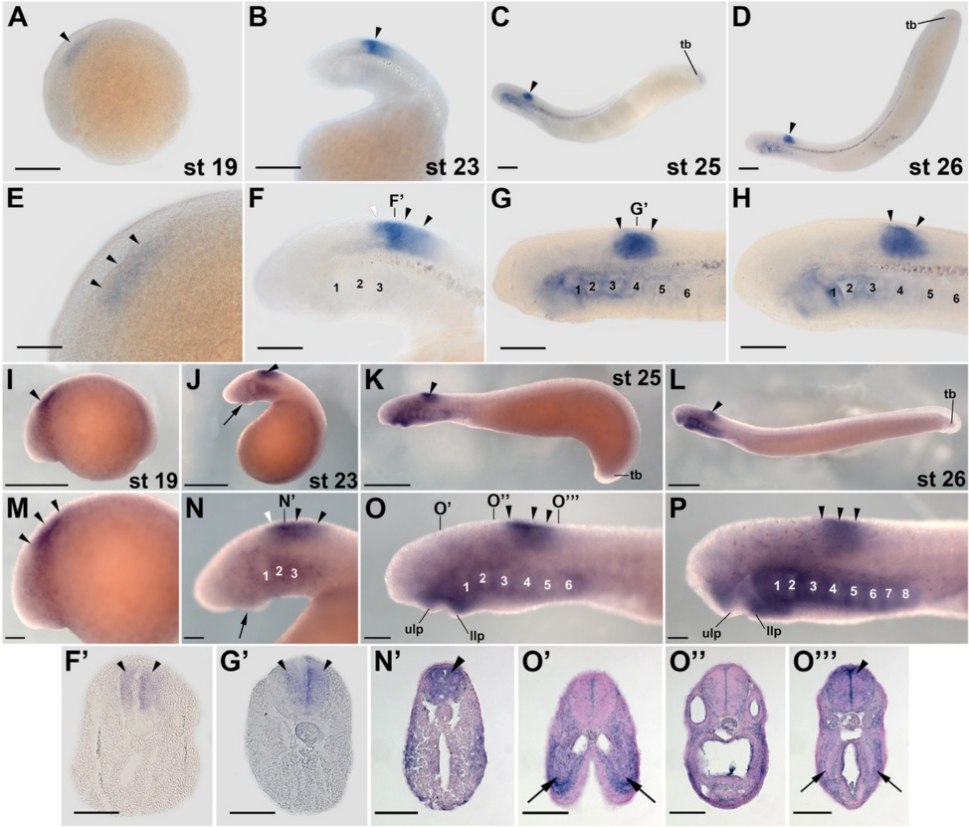
Expression of the retinoic acid receptor (*rar*) gene *rar4* during development of the European river lamprey, *Lampetra fluviatilis*, and of the sea lamprey, *Petromyzon marinus*. The expression of *L. fluviatilis rar4* was characterized by *in situ* hybridization in embryos at stages (st) 19 **(A)** and **(E)**, 23 **(B)** and **(F)**, 25 **(C)** and **(G)**, as well as 26 **(D)** and **(H)**. Higher magnifications of the anterior regions of embryos at stages 19, 23, 25, and 26 are shown in **(E)**, **(F)**, **(G)**, and **(H)**, respectively. Cross sections of the embryos at stages 23 **(F’)** and 25 **(G’)** are through the anterior spinal cord. The expression of *P. marinus rar4* was characterized by *in situ* hybridization in embryos at stages 19 **(I)** and **(M)**, 23 **(J)** and **(N)**, 25 **(K)** and **(O)**, as well as 26 **(L)** and **(P)**. Higher magnifications of the anterior regions of embryos at stages 19, 23, 25, and 26 are shown in **(M)**, **(N)**, **(O)**, and **(P)**, respectively. The cross section of the embryo at stage 23 is through the anterior spinal cord **(N’)** and those of the embryo at stage 25 are through the mouth region **(O’)**, otic vesicle **(O”)**, and posterior pharynx **(O''')**. All embryos are oriented with anterior to the left. White arrowheads indicate hindbrain expression and black arrowheads expression in the spinal cord. Arrows in **(O''')** show pharyngeal expression, while arrows in **(J)**, **(N)**, as well as in **(O’)** highlight mandibular expression. Scale bars for *L. fluviatilis*: 100 μm for **(A)**, **(B)**, **(C)**, and **(D)**; 50 μm for **(E)**, **(F)**, **(G)**, **(H)**, **(F’)**, and **(G’)**. Scale bars for *P. marinus*: 500 μm for **(I)**, **(J)**, **(K)**, and **(L)**; 100 μm for **(M)**, **(N)**, **(O)**, **(P)**, **(N’)**, **(O’)**, **(O”)**, and **(O”’)**. Abbreviations: llp, lower lip; tb, tail bud; ulp, upper lip; 1 to 8, pharyngeal pouches 1 to 8.

Based on the results of previous phylogenetic analyses [31], the lamprey *rar1* and *rar4* genes probably belong to the gnathostome *rarγ* subtype and may have arisen from a lamprey-specific duplication that, according to the results of our phylogenetic inference (Figure 1), probably occurred after the divergence from the hagfish lineage. Despite their relative phylogenetic proximity, *rar1* and *rar4* exhibit very different expression patterns during lamprey development (Figures 2 and 3). In *L. fluviatilis* embryos at stage 19, *rar1* expression is detected in the hindbrain and anterior spinal cord, with expression in the hindbrain being less conspicuous than in the spinal cord (Figure 2A,E). In the course of development (that is, at stages 23 and 25), *L. fluviatilis rar1* expression splits into an anterior domain, comprising rhombomeres 1 and 2, and a posterior domain, which includes the anterior spinal cord (Figure 2B,C,F,G). Interestingly, at least the posteriormost neural expression of *rar1* seems to be slightly more conspicuous dorsally (Figure 2G”). At stage 26, expression in the anterior hindbrain is lost, while *rar1* transcripts are still detectable in the spinal cord (Figure 2D, H).

Outside the CNS, *rar1* is dynamically expressed in several structures of the developing *L. fluviatilis* embryo. For example, at stage 19, expression of *rar1* is detected in the presumptive pharyngeal region (Figure 2A,E). Pharyngeal expression of *rar1* becomes more conspicuous at stage 23, especially in the mandibular region (that is, in the upper and lower lips). Later on, at stages 25 and 26, *rar1* is prominently expressed in the pharynx (Figure 2G’) as well as in the upper and lower lips of the mouth (Figure 2C,D,G,H). This widespread expression of *rar1* in the pharyngeal region is suggestive of a role for RA signaling in the patterning and specification of the developing lamprey pharynx, as has recently been proposed [24]. At stages 25 and 26, *rar1* is further expressed in the tail bud (Figure 2C,D) and, at least at stage 25, in the otic vesicle (Figure 2C,D,G,H).

Comparisons of *rar1* expression between the three different lamprey species, that is, between *L. fluviatilis* (Figure 2A-H,G’,G”), *P. marinus* (Figure 2I-P,N’,O’,O”,O”’), and *L. japonicum* (Figure 2Q-S,S’,S”,S”’,S””), reveal that the expression patterns are generally well conserved. At corresponding developmental stages, the main differences are discernable in the otic vesicle, where *rar1* expression is conspicuous in *L. fluviatilis* and *P. marinus* and likely absent in *L. japonicum*. Furthermore, *rar1* is also abundantly expressed in the pharynx of both *L. fluviatilis* and *P. marinus* embryos, with the notable difference of *L. fluviatilis* turning on the pharyngeal expression of this gene earlier than *P. marinus*. Interestingly, in *L. japonicum* the pharyngeal signal seems to be restricted exclusively to the buccal cavity. Finally, tail bud-associated expression of *rar1* is detectable in *L. fluviatilis*, inconspicuous in *P. marinus,* and likely absent in *L. japonicum*.

In contrast to the other lamprey *rar* genes, expression of *rar4* has only been assessed in *L. fluviatilis* and *P. marinus* (Figure 3). In *L. fluviatilis*, *rar4* expression, from stage 19 through 26, is almost exclusively restricted to the developing CNS, conspicuously in the anterior spinal cord and transiently, at stage 23, in the posteriormost hindbrain (Figure 3A-H,F’,G’). Cross sections indicate that the gene is homogeneously expressed along the dorsoventral axis of the spinal cord, at least from stage 23 through 26 (Figure 3F’,G’). The only other tissues that might express *rar4* at the assayed stages are the most posterior hindbrain at stages 19 and 23 as well as the anterior pharyngeal gill pouches and the tail bud at stages 25 and 26 (Figure 3C,D,G,H). Of note, the expression domains of *rar4* do not seem to change significantly during lamprey development and are thus remarkably stable over time (Figure 3A-D). The expression of *rar4* in *P. marinus* (Figure 3I-P,N’,O’,O”,O”’) generally resembles that of *L. fluviatilis* and suggests that the expression patterns between both lamprey species are well conserved. Slight differences can be observed in the onset of pharyngeal expression, which seems to be advanced in *P. marinus*. Thus, in this species, very weak pharyngeal expression can already be observed at stage 23 (Figure 3J,N), while it only appears in *L. fluviatilis* at stage 25 (Figure 3C,G).

Taken together, although *rar1* and *rar4* exhibit partially overlapping expression domains during lamprey development, most conspicuously in the anterior CNS, *rar1* expression is dynamic, whereas *rar4* expression is stable through embryogenesis. This difference suggests that, following the lineage-specific gene duplication, the regulatory regions of *rar1* and *rar4* have evolved distinct sets of transcriptional control elements.

In other vertebrates, genes of the *rarγ* subtype are expressed in various tissues in the course of development. For example, in frog and mouse embryos, *rarγ* is expressed in the tail bud and the pharynx as well as in specific regions of the developing brain [19]. In contrast, developmental expression of *rarγa* and *rarγb* in zebrafish is strikingly different from *rarγ* expression in other gnathostomes as well as from that described here for lampreys. Thus, zebrafish *rarγa* is expressed in a dynamic rhombomere-specific pattern in the anterior CNS, whereas *rarγb* expression is not at all detected in hindbrain structures [17,46]. Although certainly not the result of the same gene duplication event, the divergence of the *rarγa* and *rarγb* expression patterns in zebrafish is nonetheless reminiscent of the situation of the *rar1* and *rar4* expression patterns in lampreys. Following the gene duplication, the regulatory regions of zebrafish *rarγa* and *rarγb* thus very likely accumulated mutations that led to the acquisition of distinct sets of developmental expression domains, which in turn resulted in the preservation of both duplicates in the genome [47].

### Expression of lamprey *rar2* genes during development

**Figure 4:**
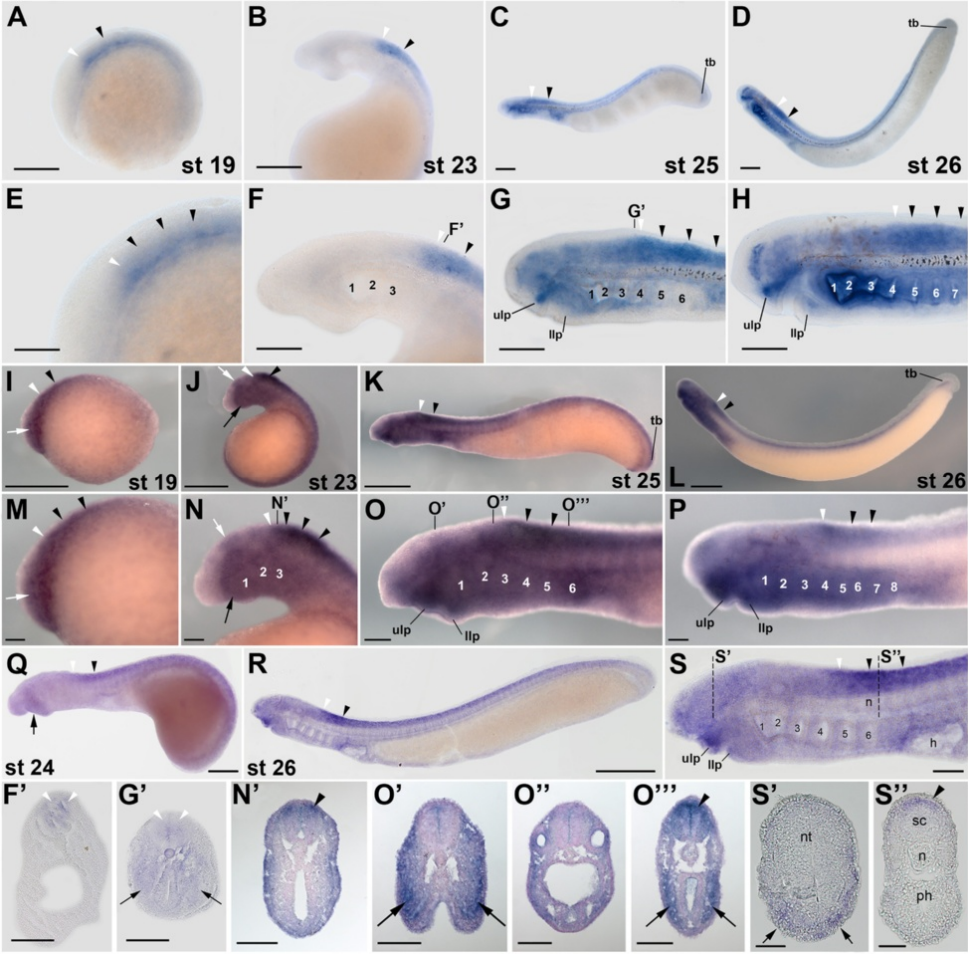
Expression of the retinoic acid receptor (*rar*) gene *rar2* during development of the European river lamprey, *Lampetra fluviatilis*, of the sea lamprey, *Petromyzon marinus*, and of the Japanese lamprey, *Lethenteron japonicum*. The expression of *L. fluviatilis rar2* was characterized by *in situ* hybridization in embryos at stages (st) 19 **(A)** and **(E)**, 23 **(B)** and **(F)**, 25 **(C)** and **(G),** as well as 26 **(D)** and **(H)**. Higher magnifications of the anterior regions of embryos at stages 19, 23, 25, and 26 are shown in **(E)**, **(F)**, **(G)**, and **(H)**, respectively. The cross sections of the embryo at stage 23 **(F’)** and of the embryo at stage 25 **(G’)** are through the level of the posteriormost hindbrain. The expression of *P. marinus rar2* was characterized by *in situ* hybridization in embryos at stages 19 **(I)** and **(M)**, 23 **(J)** and **(N)**, 25 **(K)** and **(O)**, as well as 26 **(L)** and **(P)**. Higher magnifications of the anterior regions of embryos at stages 19, 23, 25, and 26 are shown in **(M)**, **(N)**, **(O)**, and **(P)**, respectively. The cross section of the embryo at stage 23 is through the anterior spinal cord **(N’)** and those of the embryo at stage 25 are through the mouth region **(O’)**, otic vesicle **(O”)**, and posterior pharynx **(O''')**. The expression of *L. japonicum rar2* was analyzed by *in situ* hybridization in embryos at stages 24 **(Q)** as well as 26 **(R)** and **(S)**. A higher magnification of the anterior region of the embryos at stage 26 is shown in **(S)**. Cross sections of the embryo at stage 26 are through the mouth region **(S’)** and anterior spinal cord **(S”)**. The exact anteroposterior levels of the sections shown in **(S’)** and **(S”)** are indicated by dotted lines in **(S)**. All embryos are oriented with anterior to the left. White arrowheads indicate hindbrain expression and black arrowheads expression in the spinal cord. Black arrows in **(G’)** and **(O''')** show pharyngeal expression, while black arrows in **(J)**, **(N)**, and **(Q)**, as well as in **(O’)** and **(S’)** highlight mandibular expression. White arrows mark an inconspicuous *rar2* expression domain in the anterior *P. marinus* hindbrain at stages 19 and 23. Scale bars for *L. fluviatilis*: 100 μm for **(A)**, **(B)**, **(C)**, and **(D)**; 50 μm for **(E)**, **(F)**, **(G)**, **(H)**, **(F’)**, and **(G’)**. Scale bars for *P. marinus*: 500 μm for **(I)**, **(J)**, **(K)**, and **(L)**; 100 μm for **(M)**, **(N)**, **(O)**, **(P)**, **(N’)**, **(O’)**, **(O”)**, and **(O''')**. Scale bars for *L. japonicum*: 500 μm for **(Q)**; 1,000 μm for **(R)**; 100 μm for **(S)**; 50 μm for **(S’)** and **(S”)**. Abbreviations: h, heart; llp, lower lip; n, notochord; nt, neural tube; ov, otic vesicle; ph, pharynx; sc, spinal cord; tb, tail bud; ulp, upper lip; 1 to 8, pharyngeal pouches 1 to 8.

Transcripts of *L. fluviatilis rar2*, a possible ortholog of the gnathostome *rarβ* subtype [31], are detectable by *in situ* hybridization from stage 19 onwards (Figure 4A,E). Interestingly, the domains of *rar2* expression in the *L. fluviatilis* CNS do not significantly change between stages 19 and 26. Thus, *rar2* is expressed all along the CNS, with an anterior limit of expression in the posterior hindbrain at the level of rhombomere 6. In addition, the neural expression of *rar2* is homogenous along the dorsoventral axis, as shown in cross sections at stage 23 (Figure 4B,F,F’). These characteristics of the expression of the lamprey *rar2* are reminiscent of *rarβ* expression in frogs and mice, where *rarβ* is conspicuously detectable in the spinal cord and characterized by an anterior limit of expression in the posterior hindbrain [19]. In addition to the spinal cord and the posterior hindbrain, at stages 25 and 26, *L. fluviatilis rar2* is also prominently expressed in the upper and very weakly in the lower lip of the mouth as well as in the pharyngeal pouches (Figure 4C,D,G,H), which is also comparable to the expression of *rarβ* in frogs and mice [19]. While, in stage 25 embryos, the pharyngeal expression of *rar2* is initially separated into two distinct domains, one located anteriorly and one posteriorly in the pharynx, the *rar2* signal subsequently expands and becomes detectable throughout the pharynx at stage 26 (Figure 4C,D,G,H,G’). This conspicuous expression of *rar2* in the pharynx further supports the notion that RA signaling might be required for pharyngeal patterning and specification in the lamprey embryo [24]. Finally, at stages 25 and 26, *rar2* is also expressed inconspicuously in the *L. fluviatilis* tail bud (Figure 4C,D).

Comparisons of *rar2* expression between *L. fluviatilis* (Figure 4A-H,F’,G’), *P. marinus* (Figure 4I-P,N’,O’,O”,O”’), and *L. japonicum* (Figure 4Q-S,S’,S”) indicate that the overall patterns are very well conserved, with the main differences being detectable in the pharynx, where both *L. fluviatilis* and *P. marinus rar2* are expressed in the pharyngeal pouches, while the *L. japonicum rar2* signal seems to be limited to the pharyngeal territory just anterior to the heart. Intriguingly, contrary to the situation of *L. fluviatilis* and *P. marinus rar1*, but similar to the situation of *L. fluviatilis* and *P. marinus rar4*, the pharyngeal expression of *L. fluviatilis rar2* turns on only after that of *P. marinus rar2*. Further *rar2* expression differences between the three lamprey species include an inconspicuous domain in the anterior hindbrain detectable exclusively in *P. marinus* at stages 19 and 23, a stronger mandibular signal in *P. marinus,* as well as the tail bud-associated expression domain of this gene, which is obvious in *P. marinus*, weak in *L. fluviatilis*, and possibly absent in *L. japonicum*.

It has previously been proposed that members of the *rarβ* subtype have retained expression patterns that resemble the ancestral vertebrate condition [19]. This hypothesis is based on the observation that the domains of both frog and mouse *rarβ* expression are comparable to those of the single amphioxus *rar*. Furthermore, the amphioxus RAR ligand-binding pocket exhibits a ligand-binding selectivity that is of a RARβ type, which was thus proposed to be a chordate synapomorphy [19]. The patterns of lamprey *rar2* expression, reported here, closely resemble those of both frog and mouse *rarβ* as well as those of the single amphioxus *rar*. These data thus seem to confirm the hypothesis that, of the three gnathostome *rar* subtypes, the developmental expression, and possibly function, of *rarβ* genes most closely approximate the ancestral vertebrate condition. Along these lines, it is interesting to note that the zebrafish genome does not encode any *rarβ* ortholog, while the medaka fish genome contains two *rarβ* genes [48], indicating that the *rarβ* subtype was specifically lost in the lineage leading to extant zebrafish [16].

### Expression of lamprey *rar3* genes during development

**Figure 5:**
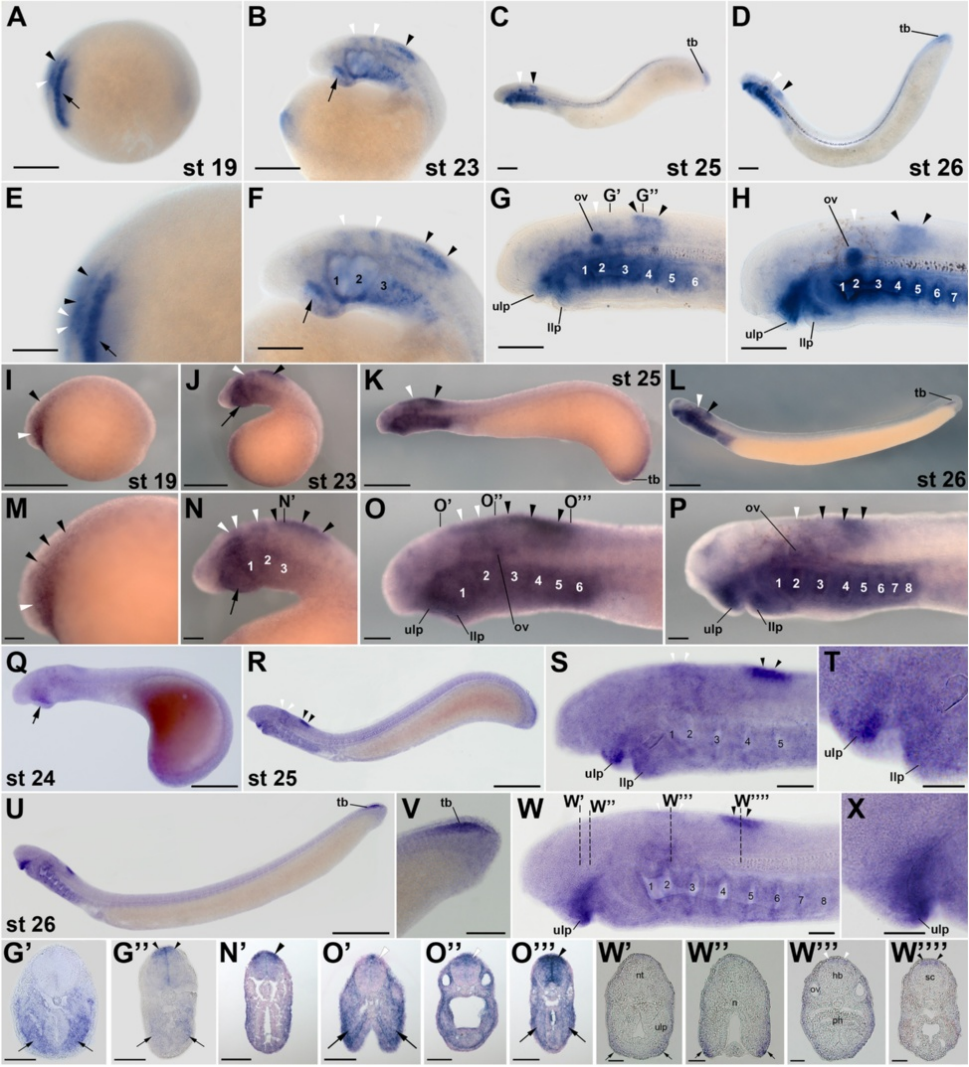
Expression of the retinoic acid receptor (*rar*) gene *rar3* during development of the European river lamprey, *Lampetra fluviatilis*, of the sea lamprey, *Petromyzon marinus*, and of the Japanese lamprey, *Lethenteron japonicum*. The expression of *L. fluviatilis rar3* was characterized by *in situ* hybridization in embryos at stages (st) 19 **(A)** and **(E)**, 23 **(B)** and **(F)**, 25 **(C)** and **(G)**, as well as 26 **(D)** and **(H)**. Higher magnifications of the anterior regions of embryos at stages 19, 23, 25, and 26 are shown in **(E)**, **(F)**, **(G)**, and **(H)**, respectively. Cross sections of the embryo at stage 25 are through the posterior hindbrain **(G’)** and the anterior spinal cord **(G”)**. The expression of *P. marinus rar3* was characterized by *in situ* hybridization in embryos at stages 19 **(I)** and **(M)**, 23 **(J)** and **(N)**, 25 **(K)** and **(O)**, as well as 26 **(L)** and **(P)**. Higher magnifications of the anterior regions of embryos at stages 19, 23, 25, and 26 are shown in **(M)**, **(N)**, **(O),** and **(P)**, respectively. The cross section of the embryo at stage 23 is through the anterior spinal cord **(N’)** and those of the embryo at stage 25 through the mouth region **(O’)**, otic vesicle **(O”)**, and posterior pharynx **(O”’)**. The expression of *L. japonicum rar3* was analyzed by *in situ* hybridization in embryos at stages 24 **(Q)**, 25 **(R)** and **(S)**, as well as 26 **(U)** and **(W)**. Higher magnifications of the anterior regions of embryos at stages 25 and 26 are shown in **(S)** and **(W)**, respectively. The mouth regions of embryos at stages 25 and 26 are further magnified in **(T)** and **(X)**, respectively. A magnification of the tail of the stage 26 embryo is shown in **(V)**. Cross sections of the embryo at stage 26 are through the mouth region **(W’)** and **(W”)**, the posterior hindbrain **(W''')**, and the anterior spinal cord **(W””)**. The exact anteroposterior levels of the sections shown in **(W’)**, **(W”)**, **(W”’)**, and **(W'''')** are indicated by dotted lines in **(W)**. All embryos are oriented with the anterior to the left. White arrowheads indicate hindbrain expression and black arrowheads expression in the spinal cord. Arrows in **(A)**, **(E)**, **(G’)**, **(G”)**, and **(O”’)** show pharyngeal expression, while arrows in **(B)**, **(F)**, **(J)**, **(N)**, **(Q)**, **(O’)**, **(W’)**, and **(W”)** highlight mandibular expression. Scale bars for *L. fluviatilis*: 100 μm for **(A)**, **(B)**, **(C)**, and **(D)**; 50 μm for **(E)**, **(F)**, **(G)**, **(H)**, **(G’)**, and **(G”)**. Scale bars for *P. marinus*: 500 μm for **(I)**, **(J)**, **(K)**, and **(L)**; 100 μm for **(M)**, **(N)**, **(O)**, **(P)**, **(N’), (O’)**, **(O”)**, and **(O''')**. Scale bars for *L. japonicum*: 500 μm for **(Q)** and **(V)**; 1,000 μm for **(R)** and **(U)**; 100 μm for **(S)** and **(W)**; 50 μm for **(T)**, **(X)**, **(W’)**, **(W”)**, **(W”’)**, and **(W””)**. Abbreviations: hb, hindbrain; llp, lower lip; n, notochord; nt, neural tube; ov, otic vesicle; ph, pharynx; sc, spinal cord; tb, tail bud; ulp, upper lip; 1 to 8, pharyngeal pouches 1 to 8.

The lamprey *rar3* gene is a likely ortholog of the gnathostome *rarα* subtype [31], and its expression in *L. fluviatilis*, like the one of *rar2*, is already detectable by *in situ* hybridization in stage 19 embryos. Thus, in stage 19 *L. fluviatilis* embryos, *rar3* is expressed in neural tissues, more specifically in the presumptive hindbrain and anterior spinal cord, as well as in the future pharynx (Figure 5A,E). Later in development, at stage 23, two distinct domains of *L. fluviatilis rar3* expression are observable in the anterior CNS: the first one in the hindbrain, in rhombomeres 4 and 5, and the second one in the anterior spinal cord (Figure 5B,F). These separate domains likely arose from the unique signal observed earlier in development, through partitioning during elongation of the embryo. In addition to its expression in neural tissues, at stage 23, *L. fluviatilis rar3* is also detectable in the upper and lower lips as well as in the pharynx, both anteriorly in differentiated and posteriorly in presumptive pharyngeal pouches (Figure 5B,F).

At stages 25 and 26, expression of *L. fluviatilis rar3* in the anterior CNS does not significantly change from its expression at stage 23 (Figure 5C,D,G,H). Thus, both the domains in rhombomeres 4 and 5 and in the anterior spinal cord are maintained dorsally in the CNS (Figure 5G”), even though expression in the hindbrain is much less conspicuous at stages 25 and 26, when compared to the signal at stage 23. Furthermore, *L. fluviatilis rar3* expression is strongly induced in the newly formed otic vesicle at stage 25, which closely correlates with the differentiation of this anatomical structure derived from the ectoderm, and the expression is maintained at stage 26 (Figure 5C,D,G,H). The observation of *rar* expression in the lamprey otic vesicle is in accordance with results obtained in other vertebrate models, where *rar* genes are specifically expressed in the developing ear and contribute to a time- and space-dependent activation of the RA signaling cascade [20]. Consistent with the signals detected at earlier stages, at stages 25 and 26, *L. fluviatilis rar3* is still strongly expressed in the pharyngeal region, in all pharyngeal pouches (Figure 5C,D,G,H). This pharyngeal expression is clearly visible in cross sections of stage 25 larvae (Figure 5G’,G”). Moreover, *L. fluviatilis rar3* is also detectable in the mouth region at stages 25 and 26, both in the upper and lower lips (Figure 5C, D, G, H). As discussed above for *rar1* and *rar2*, the pharyngeal expression of *rar3* is highly suggestive of a direct implication of RA signaling in patterning and specification of the developing lamprey pharynx [24].

The expression of *rar3* is quite well conserved between *L. fluviatilis* (Figure 5A-H,G’,G”), *P. marinus* (Figure 5I-P,N’,O’,O”,O”’), and *L. japonicum* (Figure 5Q-X,W’,W”,W”’,W””). As for the other lamprey *rar* genes, the main differences are detectable in the pharynx. Thus, while *rar3* is clearly detectable in the pharyngeal region of both *L. fluviatilis* and *P. marinus*, the *L. japonicum* pharynx does not seem to express this gene. In *P. marinus*, *rar3* was shown to be the only *rar* gene expressed in the pharyngeal endoderm [24], which is consistent with our results from the three lamprey species. In addition to the pharynx, lamprey *rar3* genes also seem to be differentially expressed in the developing otic vesicle. Thus, while the gene is conspicuously expressed in the otic vesicle of *L. fluviatilis* embryos, its expression in this structure is only weak in *P. marinus* and undetectable by *in situ* hybridization in developing *L. japonicum*.

When compared to *rarα* expression in other vertebrates, the lamprey *rar3* pattern shows both similarities and significant differences. For example, in the frog and the mouse, *rarα* is broadly expressed in various embryonic tissues during development, including the neuroectoderm and the mesenchyme of the head [19]. The expression of *rar3* in lampreys, with specific domains in CNS, pharynx, and otic vesicle, more closely resembles the situation in zebrafish, where both *rarαa* and *rarαb* are expressed in distinct regions of the developing embryo [15,17,46]. Even if only the expression domains of gnathostome *rarα* subtype genes in the CNS are compared, despite some conserved domains of expression (for example, in the posterior rhombomeres and the anterior spinal cord), the overall spatiotemporal dynamics of gene expression seem to vary significantly between different species [17,19,20]. Taken together, these comparisons suggest that, in the course of vertebrate evolution, the developmental expression of *rarα* paralogs has been subjected to lineage-specific modifications.

## Conclusions

In this study, we have identified four *rar* genes (*rar1*, *rar2*, *rar3*, and *rar4*) in three lamprey species, the European river lamprey, *L. fluviatilis*, the sea lamprey, *P. marinus*, and the Japanese lamprey, *L. japonicum*, and subsequently analyzed their phylogenetic position in a tree of the RAR subfamily of the nuclear hormone receptors. We further assessed the developmental expression of the *rar* genes in these three lamprey species.

Our results are compatible with the notion that lamprey genomes encode orthologs of each of the three gnathostome *rar* subtypes (*rarα*, *rarβ*, and *rarγ*), hence supporting the hypothesis that lampreys have also undergone the two rounds of WGD that occurred early in vertebrate evolution [31]. Furthermore, the work reported here suggests that lampreys possess two *rar* genes (*rar1* and *rar4*) that duplicated after the split of the lamprey and hagfish lineages.

**Figure 6:**
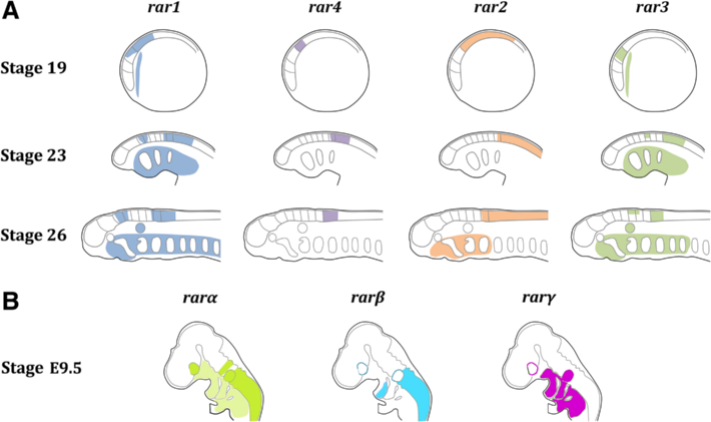
Diagrammatic summary of retinoic acid receptor (*rar*) expression during development of the European river lamprey, *Lampetra fluviatilis*, and of the house mouse, *Mus musculus*. Indicated, in **(A)**, are the expression domains of the four *L. fluviatilis rar* genes (*rar1*, *rar2*, *rar3*, and *rar4*) at developmental stages 19, 23, and 26 and, in **(B)**, of the three *M. musculus rar* genes (*rarα*, *rarβ*, and *rarγ*) at developmental stage E9.5 [20]. Comparisons focus on gene expression in central nervous system (CNS), pharyngeal region, as well as optic and otic territories. See main text for detailed description of *rar* expression in *L. fluviatilis*. In mouse embryos at stage E9.5, *rarα* is expressed in the CNS (in rhombomere 4 and in spinal cord and posterior hindbrain up to the rhombomere 6/7 boundary), in optic and otic territories, as well as broadly, but inconspicuously, in the pharyngeal region. At E9.5, *rarβ* is expressed in the CNS (spinal cord and posterior hindbrain up to the rhombomere 6/7 boundary), in the posterior portion of the first pharyngeal arch, as well as in tissues surrounding the optic and otic vesicles. At E9.5, *rarγ* is not transcribed in the CNS, but is broadly expressed in the pharyngeal region as well as the otic territory and is further detectable in tissues surrounding the optic vesicle.

The *in situ* hybridization experiments indicate that lamprey *rar* genes are expressed in very specific spatiotemporal patterns during development (Figure 6). In particular, the expression domains of the different *rar* genes in the anterior CNS are highly regionalized, and partially overlapping expression of one or several *rar* genes are observable in different regions of the CNS, such as the hindbrain and the anterior spinal cord. This combinatorial expression of *rar* genes in different domains of the anterior CNS during neurulation hence creates a ‘RAR code’ defining specific regions of the developing lamprey brain (Figure 6). In addition to the CNS, a ‘RAR code’ might also be observable in the developing pharyngeal region (Figure 6), although characterized by a different spatiotemporal combination of *rar* expression.

Given that RA treatments of developing lamprey embryos induce severe rostral truncations, in particular in CNS and pharynx [22], it is very appealing to speculate that the ‘RAR codes’ in these two tissues are functionally required to ensure proper regional patterning and tissue specification. Indeed, in zebrafish, *rarαa*, *rarαb*, *rarγa*, and *rarγb* operate combinatorially to pattern the hindbrain, pharyngeal arches, and pectoral fins [46]. Furthermore, several studies have shown that RA signaling is required for the establishment of another gene expression ‘code’ during chordate development: the so-called ‘Hox code’ that is crucial for anteroposterior patterning of the embryo [3-5,49] and that has also been described in the lamprey embryo [50,51]. Given that *hox* genes figure prominently amongst the described direct targets of RAR-dependent signaling [3-5], it is very tempting to speculate that at least some of the functional readout of the lamprey ‘RAR code’ is directly translated into the lamprey ‘Hox code’ and that this relationship between the ‘RAR code’ and the ‘Hox code’ has evolved in the last common ancestor of extant vertebrates following the two rounds of WGD.

Comparisons of the developmental expression of *rar* genes between vertebrates (Figure 6) reveal that, while the spatiotemporal patterns of *rarα* and *rarγ* subtype genes display a relatively high degree of diversity between species, the developmental expression of *rarβ* subtype genes is more conserved, at least between lampreys, frogs, and mice [19]. Previous studies have proposed that vertebrate *rarβ* genes have retained ancestral chordate characters, both in terms of developmental gene expression and ligand-binding properties of the receptor [19]. Our analyses of the expression of *rar* genes during lamprey development support this hypothesis (Figure 6), which was initially elaborated based on the characterization of the *rar* gene of the invertebrate chordate amphioxus. It is thus likely that, following the two rounds of WGD, which occurred before the cyclostome-gnathostome split, the members of the three *rar* subtypes diversified their developmental expression and functions, with one subtype (*rarβ*) retaining ancestral characteristics and the other two subtypes (*rarα* and *rarγ*) acquiring novel features. In this context, the emergence of ‘RAR codes’ in different embryonic tissues may have resulted in the elaboration of new regulatory circuitries supporting the evolution of novel developmental features and hence of morphological innovations.

## Additional files

Additional file 1: Figure S1. Precise mapping of retinoic acid receptor (*rar*) expression in the European river lamprey, *Lampetra fluviatilis*, by one color double *in situ* hybridization. The top panel shows expression of each one of the four *rar* genes in *L. fluviatilis* embryos at stage 23. The middle panel displays expression of *krox20* in rhombomeres 3 and 5 in a *L. fluviatilis* embryo at stage 23. The bottom panel shows expression of each one of the four *rar* genes together with that of *krox20* in *L. fluviatilis* embryos at stage 23. Embryos are oriented with anterior to the left. Scale bars: 50 μm. Additional file 2: Figure S2. Precise mapping of retinoic acid receptor (*rar*) expression in the European river lamprey, *Lampetra fluviatilis*, by immunohistochemistry. The top panel shows the expression of *rar4* in a *L. fluviatilis* embryo at stage 25. The bottom panel displays a *L. fluviatilis* embryo at stage 25 labeled with an antibody directed against acetylated tubulin (Ac-tubulin). The fluorescent signal is detectable in cranial nerves and specific regions of the brain. Embryos are oriented with anterior to the left. Scale bars: 50 μm.

## Abbreviations

AP: alkaline phosphatase
bp: base pairs
cDNA: complementary deoxyribonucleic acid
CHAPS: 3-[(3-cholamidopropyl)dimethylammonio]-1-propanesulfonate
CNS: central nervous system
DAB: 3,3′-diaminobenzidine
DIG: digoxigenin
DMSO: dimethylsulfoxide
EDTA: ethylenediaminetetraacetic acid
Fab: fragment antigen-binding
hox: homeobox
HRP: horseradish peroxidase
IgG: immunoglobulin G
MABT: maleic acid buffer (pH 7.5), 0.1% Tween 20
MgCl_2_: magnesium chloride
ML: maximum likelihood
MMR: Marc’s modified Ringer’s
NaCl: sodium chloride
NTE: 10 mM Tris–HCl (pH 8), 0.5 M NaCl, 1 mM EDTA
PBS: phosphate-buffered saline
PBT: PBS, 0.1% Tween 20
PCR: polymerase chain reaction
PFA: paraformaldehyde
RA: retinoic acid
RACE: rapid amplification of cDNA ends
RAR: retinoic acid receptor
RARE: retinoic acid response element
RNA: ribonucleic acid
RXR: retinoid X receptor
SSC: saline sodium citrate
Tris–HCl: 2-amino-2-(hydroxymethyl)-1,3-propanediol hydrochloride
TST: Tris-buffered saline (pH 7.6), 0.1% Tween 20
TSTd: TST, 5% DMSO
TSTM: TST, 5% DMSO, 1% periodic acid, 5% nonfat dry milk
UTP: uridine triphosphate
WGD: whole genome duplication
