# Supplementary figures and images for: Evolution of retinoic acid receptors in chordates: insights from three lamprey species, Lampetra fluviatilis, Petromyzon marinus, and Lethenteron japonicum
Source: EvoDevo. 2015 May 7;6:18. doi: 10.1186/s13227-015-0016-4 (PMC4432984; doi:10.1186/s13227-015-0016-4)

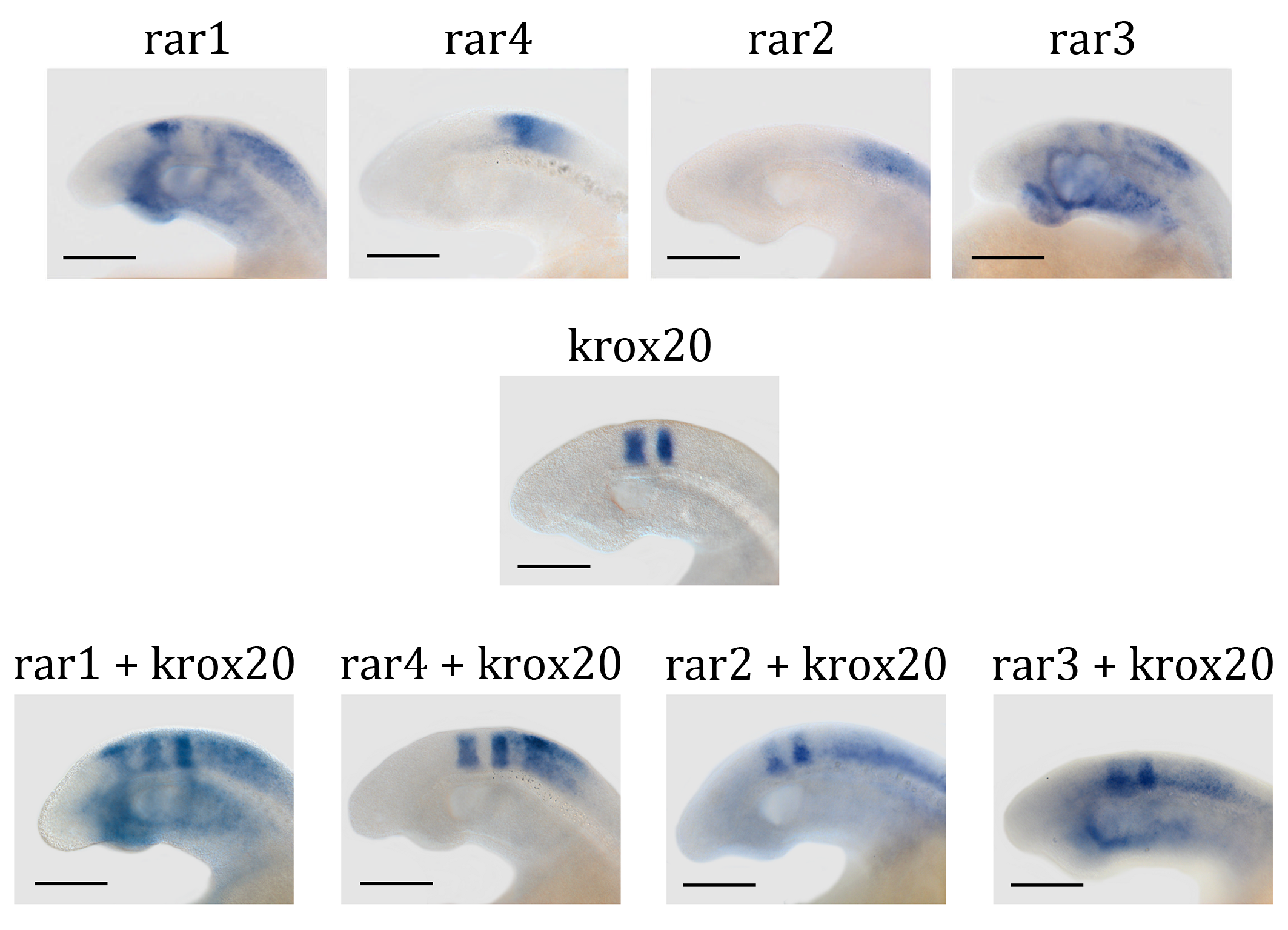

Supplement: Additional file 1: Figure S1. — Precise mapping of retinoic acid receptor (rar) expression in the European river lamprey, Lampetra fluviatilis, by one color double in situ hybridization. The top panel shows expression of each one of the four rar genes in L. fluviatilis embryos at stage 23. The middle panel displays expression of krox20 in rhombomeres 3 and 5 in a L. fluviatilis embryo at stage 23. The bottom panel shows expression of each one of the four rar genes together with that of krox20 in L. fluviatilis embryos at stage 23. Embryos are oriented with anterior to the left. Scale bars: 50 μm. [file 13227_2015_16_MOESM1_ESM.tiff]

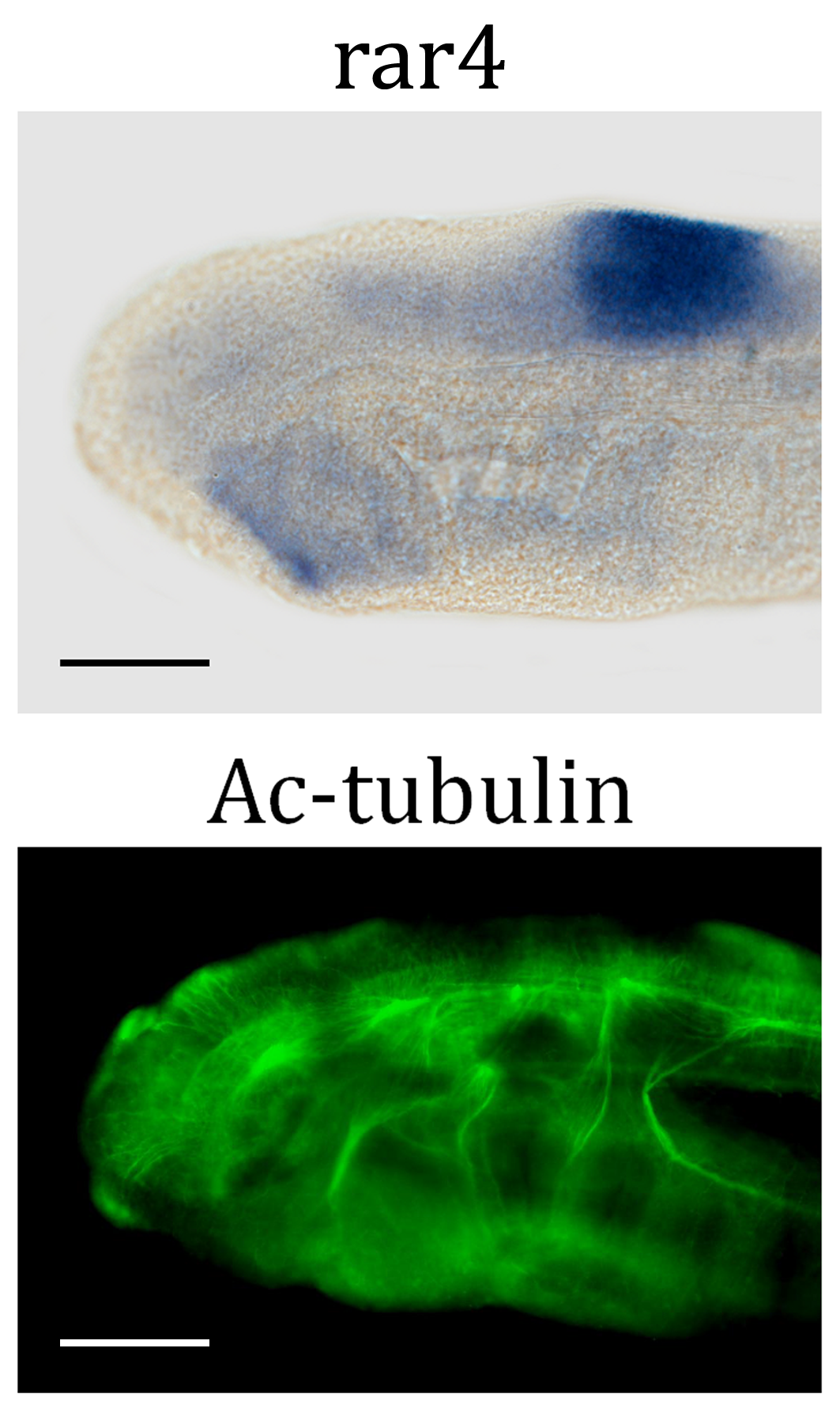

Supplement: Additional file 2: Figure S2. — Precise mapping of retinoic acid receptor (rar) expression in the European river lamprey, Lampetra fluviatilis, by immunohistochemistry. The top panel shows the expression of rar4 in a L. fluviatilis embryo at stage 25. The bottom panel displays a L. fluviatilis embryo at stage 25 labeled with an antibody directed against acetylated tubulin (Ac-tubulin). The fluorescent signal is detectable in cranial nerves and specific regions of the brain. Embryos are oriented with anterior to the left. Scale bars: 50 μm. [file 13227_2015_16_MOESM2_ESM.tiff]
